# Investigation of magnesium aluminometasilicate (Neusilin US2) based surface solid dispersion of sorafenib tosylate using QbD approach: *In vitro* and *in vivo* pharmacokinetic study

**DOI:** 10.5599/admet.2338

**Published:** 2024-08-09

**Authors:** Bijoy Kumar Panda, Bothiraja Chellampillai, Sharad Ghodake, Ashwin J. Mali, Ravindra Kamble

**Affiliations:** 1 Department of Pharmacy Practice, Krishna Institute of Pharmacy, Krishna Vishwa Vidyapeeth (Deemed to be University), Karad, Maharashtra, India; 2 Department of Pharmaceutics, Goa College of Pharmacy, Goa University, Panaji, Goa, India; 3 Department of Pharmaceutics, Poona College of Pharmacy, Bharati Vidyapeeth (Deemed to be University), Pune, Maharashtra, India

**Keywords:** Neusilin^®^ US2, Quality by design, sorafenib, pharmacokinetic analysis

## Abstract

**Background and purpose:**

Sorafenib tosylate (SFN), a potent multikinase inhibitor, is used for the treatment of various cancers. However, it shows limited therapeutic applications due to its poor biopharmaceutical properties. The aim of the present investigation is to develop surface solid dispersion (SSD) of SFN using adsorbent to improve its solubility, bioavailability and therapeutic efficacy.

**Experimental approach:**

The SFN-SSD was prepared by modified solvent evaporation technique using Neusilin US2 (magnesium aluminometasilicate) as an adsorbent and sodium dodecyl sulphate as a surfactant. SFN-SSD was optimized by adopting the design of experiment (DOE) using 3^2^ factorial designs and characterized in terms of in-vitro and in-vivo efficacy.

**Key results:**

The obtained SFN-SSD showed more than 20-fold improvement in SFN solubility. The FTIR depicted hydrogen bonding between SFN and Neusilin. Further, PXRD and DSC indicated the molecular dispersion of SFN to be amorphous. SFN-SSD and SFN immediate release tablets reflected cumulative release of 97.13 and 29.93 % in 1 h. The pharmacokinetics study of SFN-SSD showed 2 and 6.5-fold improvement in maximum concentration (*C*_max_,) and area under the curve (AUC_0-t_) as compared to pure SFN due to faster drug release at the absorption site.

**Conclusion:**

The study concluded that the SSD could be a scalable formulation approach and more industry-friendly technology to improve the biopharmaceutical properties of SFN.

## Introduction

**Figure 1.**
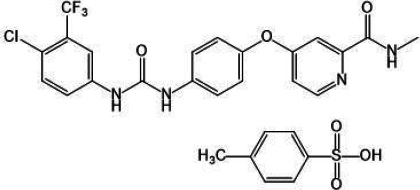
Chemical structure of sorafenib tosylate

Sorafenib tosylate (SFN), a potent multikinase inhibitor, is used for the treatment of thyroid carcinoma, advanced renal cell carcinoma and unresectable hepatocellular carcinoma [1]. SFN is a bi-aryl urea and an oral multikinase inhibitor. It consists of derivatives of bioaryl urea joined to a pyridine-2-carboxamide group, as shown in Figure 1. SFN acts by blocking the platelet-derived growth factor receptor (PDGFR), c-KIT, Flt-3, and the vascular endothelial growth factor receptor (VEGFR)-3 responsible for cancer cell growth and angiogenesis. SFN is available as film-coated tablets under the brand name Nexavar® (Bayer HealthCare Pharmaceuticals Inc.). However, it shows limited therapeutic applications due to its poor biopharmaceutical properties, such as higher lipophilicity (log *P* = 4), BCS class II (low solubility and higher permeability), solubility-limited absorption, and relative bioavailability of 38 to 49 % with the side effects of hand-foot skin responses, diarrhea, and weariness [2-4]. Various formulations such as lipid-coated nanodiamond, solid lipid nanoparticle, lipid- and poly (ethylene glycol)-coated nanoparticle, suspension, nanomatrix, and cyclodextrin-based complexes have been developed to improve its solubility, bioavailability, and therapeutic efficacy of SFN [5]. However, each formulation has its limitations. There is a need for a simple, conventional and novel formulation that could improve SFN efficacy with minimal toxicity.

Surface solid dispersion (SSD) techniques are gaining more advantages in the pharmaceutical industry to improve the solubility and bioavailability of poorly water-soluble bioactive [6-8]. In SSD, the water-insoluble pharmaceutical actives are adsorbed physically or chemically on the large surface area of the water-insoluble porous adsorbent, such as sodium starch glycolate, potato starch, pregelatinized starch, crospovidone, Neusilin® US2, Neusilin® UF, and Aerosil® 200, which have a unique higher affinity for the dissolution medium and increase the dissolution kinetics of the adsorbed drug. Neusilin® US2 (NU2) is a multifunctional excipient that solves common problems associated with tableting by facilitating a consistent flow of powder mix and optimum tablet hardness at low compression forces.

Further, NU2 is useful in protecting the active ingredient from moisture-related issues and enhancing the solubility of poorly soluble APIs. It is also responsible for converting oily or sticky APIs into free-flowing powder. We can explore a synthetic magnesium aluminometasilicate (MAS) with exceptional excipient properties for improved delivery of actives and quality of pharmaceutical preparations. Although chemically the same as traditional crystalline MAS, NU2 is structurally and functionally a very different product that serves a multitude of purposes when compared to other silicates [9]. Further, the use of additional surfactant in SSD considerably improves the dissolution rate by increasing drug wettability and a surface free energy [10-12]. Moreover, these adsorbents have tabletability properties such as direct compressibility and desirable porosity [13-16].

Considering the advantages of the SSD technique, the present study aims to develop surface solid dispersion of sorafenib tosylate (SFN-SSD) using NU2 as an adsorbent and sodium dodecyl sulphate (SDS) as a surfactant using a solvent evaporation technique for improving SFN solubility, bioavailability, and therapeutic efficacy. Initially, the screening of the adsorbents and surfactants was based on assessing the solubility improvement of SFN. Further, the design of experiment (DOE) was opted to optimize the formulation using 3^2^ factorial designs (Design Expert 11, Stat-ease) [17,18]. The optimized SFN-SSD was evaluated in terms of drug content, solubility, FTIR, DSC, SEM, and *in-vivo* pharmacokinetics studies in Wistar rats. Moreover, the optimized SFN-SSD was converted into immediate-release tablets using conventional excipients and evaluated for pharmaceutical properties.

## Materials and methods

### Materials

Sorafenib tosylate (≥99 %) was kindly provided by Cipla Ltd. Mumbai, India. Neusilin^®^ US2(≥99.9 %) was received as a gift sample from Fuji Chemical Industries Co., Ltd, Japan. Sodium dodecyl sulphate (99 %) was purchased from Merck Specialties Co Ltd, Mumbai, India. HPLC grade methanol (≥99.9 %) and acetonitrile (≥99.9%) were purchased from Ranikem, Avantor, India. All other chemicals and solvents used in this research were of analytical grade and water used was Millipore water.

### Methods

#### Screening of solvents

The saturation solubility of SFN was analysed by adding an excess amount of SFN in 2 ml of water, 0.1N HCl, phosphate buffer pH 6.8, methanol, ethanol and dimethyl sulfoxide (DMSO) solvents in Eppendorf tube and allowed to equilibrate on a mechanical shaker for 24 hours. The dispersion was filtered using a 0.45 μm syringe filter, and the amount of SFN in each solvent was determined by UV spectrometry at *λ*_max_ of 265 nm. The solvent with the maximum drug solubility was selected to prepare SSD [14].

#### Screening of adsorbents

Screening of adsorbents was done based on improvement in SFN solubility. Three silica-based porous adsorbents, Aerosil 200, Rxcipients GL 100, and NU2, were selected. SFN was dissolved in 0.2 ml of DMSO and 1 ml of ethanol, in which selected adsorbent in the ratio of 1:2 (drug: adsorbent) was added gradually with continuous trituration. The mixture was completely dried under vacuum at 60 °C. The improved solubility of SFN by each adsorbent was determined by adding an excess amount of dispersion in double distilled water (DDW).

#### Screening of surfactant

The surfactant was selected depending on the highest solubility of SFN in different concentration surfactant solutions. Briefly, an excessive amount of SFN was added to 1 ml of 1 % w/v solution of selected Poloxamer 407, Poloxamer 188, Gelucire 50/13, SDS, Transcutol P and Labrasol surfactants in Eppendorf tube [19]. Each dispersion was shaken continuously for 24 hours on a mechanical shaker, centrifuged for 15 min at 15000 rpm and filtered. The SFN concentration was determined in supernatant using UV-spectrometry at *λ*_max_ of 265 nm.

### Preparation and optimization of SFN-SSD

The SSD of SFN was prepared using a solvent evaporation technique. From the above screening analysis, the NU2 as an adsorbent, SDS as a surfactant and DMSO as a solvent were selected to prepare SSD-SFN. The concentration of NU2 and SDS were optimized by 3^2^ factorial designs using Design-Expert® software version 11, Stat-Ease, USA. As shown in Table 1, the concentration of NU2 and SDS were selected as an independent variable and the solubility of SFN as a dependent variable [20,21]. A total of nine runs were obtained by Design Expert 11. For each run, SFN concentration was constant, *i.e*. 50 mg. Equation (1) was obtained:


(1)

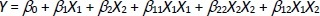



where *Y* is the measured response, *X* is the level of factors and β is the regression coefficient and *X*_1_ and *X*_2_ indicate the concentration of NU2 and SDS. A precise concentration of SFN was dissolved in 0.25 ml of DMSO in a mortar and pestle in which SDS was added with the addition of 0.5 ml of isopropyl alcohol (IPA), and the mixture was triturated for 10 mins. NU2 was added gradually to the above mixture with vigorous unidirectional trituration for another 20 minutes. The obtained SFN-SSD was dried at 60 °C for 6 hours and stored in a vacuum for further analysis.

### Characterization of SFN-SSD

#### Yield and SFN content

The dried weight of SFN-SSD was recorded as the practical yield. The SFN-SSD samples were dissolved in an appropriate amount of methanol using a cyclomixer and an ultrasonicator. The SFN content was then measured at 265 nm with a spectrophotometer (UV-530; JASCO, Japan) after making the necessary dilutions [14].

#### Saturation solubility

The solubility of SFN from SFN-SSD was analysed by adding an excess amount of SSD in 2 ml of double distilled water and kept on a mechanical shaker for 24 hours. The dispersion was filtered using a 0.45 μm syringe filter and the concentration of SFN in the filtrate was determined after suitable dilution by UV spectrometry (UV-530; JASCO, Japan) at *λ*_max_ of 265 nm [15].

#### Residual solvent content determination

The practical yield was determined by recording the dried weight of SFN-SSD. The SFN-SSD samples were dissolved in a suitable amount of methanol using a cyclomixer and an ultrasonicator. After appropriate dilutions, the SFN content was measured at 265 nm using a spectrophotometer (UV-530; JASCO, Japan).

#### Fourier transform-infrared spectroscopy

The Fourier transform-infrared (FTIR) spectra of pure SFN, NU2, physical mixture and optimized SFN-SSD were recorded using the JASCO V4100 FTIR (Tokyo, Japan). In transmission mode, the samples were triturated with IR-grade KBr and scanned throughout a 4000 to 400 cm^-1^ wavenumber range. All samples distinguishing peaks were carefully examined to identify potential interactions [16].

#### X-ray powder diffraction

The X-ray powder diffraction of Pure SFN, NU2, physical mixture and optimized SFN-SSD were assessed by powder X-ray diffractogram (PXRD) (PW 1729, Philips, Netherlands) using Cu as the anode material and a crystal graphite monochromator running at 30 kV and 30 mA. The diffractograms were produced using a 0.02626° step size and a 2 (diffraction angle) angular range of 5 to 60° [18].

#### Scanning electronic microscopy

Scanning electronic microscopy (SEM)was used to examine the surface morphology of SFN and SFN-SSD (JOEL, Japan). The samples were applied with a thin coat of gold (about 24 nm) in a vacuum to make the samples electrically conductive [16].

#### Differential scanning calorimetry

A differential scanning calorimeter (DSC) (Mettler Toledo DSC 821, Switzerland) was used to measure the thermal behaviour of the pure SFN, NU2, physical mixture and SFN-SSD. The samples (3-5 mg) were enclosed in an aluminium pan and heated at a rate of 10 °C/min while being purged with nitrogen at a flow rate of 50 mL/min. The temperature range covered was 40 to 320 °C. The temperature and enthalpy were calibrated using the industry-standard aluminium pan [18].

### Preparation of SFN-SSD and SFN immediate-release tablet

The SFN-SSD and pure SFN equivalent to 100 mg SFN immediate-release tablets were prepared by wet granulation. All ingredients were accurately weighed according to the formula given in Table 2. The SFN-SSD, croscarmellose and microcrystalline cellulose were sieved through sieve no 40 and transferred into a mortar. The PVP K30 solution in the required quantity of isopropyl alcohol was added into the mortar to coherent mass and passed through sieve no 12 to get granules. It was then kept in an oven at 120 °C for 1 hour. The dried granules were passed through sieve no. 80. The talc and magnesium stearate were added to the granules and mixed well without breaking the granules. The granules were evaluated for various flow properties such as bulk density, tapped density, Carr’s compressibility index, Hausner’s ratio and angle of repose. The tablets were punched with a 12 mm punch by the Rimek mini press (Karnavati Engineering Ltd).

### Evaluation of SFN-SSD and SFN immediate-release tablet

The tablet hardness was analysed by Incrop-PTB 311E hardness tester. Randomly, three tablets were evaluated for hardness analysis and recorded (kg/cm^2^). The thickness and diameter of the tablet were measured by Vernier calliper (Mitutoyo, USA). All measurements were done in triplicates (*n*=3). Mean thickness and diameter were reported with standard deviation. The disintegration test was performed using the Labindia-DT1000 disintegration tester in accordance with the Indian pharmacopoeia (IP) in distilled water at 37 °C. The basket's tubes were filled with the tablets and discs were placed in each basket tube. The time was noted to complete disintegration tablets. The friability test was performed as per IP, the 12 previously weighed tablets were placed in a Roche friability tester and the friability was checked at 25 rpm for 4 minutes. Friability was calculated as *F* = (*w*_i_-*w*_f_)/*w*_i_ where *w*_i_ = initial weight of tablets, *w*_f_ = final weight of tablets, where the friability of tablets less than 1 % was considered acceptable. The weight variation was calculated per IP by weighing 20 randomly selected tablets; the average weight and standard deviation were calculated.

### In vitro dissolution studies

The *in vitro* dissolution study was carried out using USP apparatus II (paddle type) dissolution apparatus (Electrolab TDT06L). The SFN-SSD and SFN instant tablet was placed in dissolution vessels containing 900 ml of 0.1 M HCl at 37 ± 0.5 °C and stirred at 75 rpm. Periodically, five millilitres of the sample were taken out from the dissolution media and replaced with fresh medium. Three duplicates of each dissolution were performed. The samples were filtered using a 0.45 μm syringe filter, and the amount of SFN dissolved in each sample was measured using UV spectroscopy at max 265 nm. Analysis of data was performed using PCP Disso v3 (Pune) software.

### Bioanalytical method of sorafenib tosylate in rat plasma

SFN was assessed in rat plasma by high-performance liquid chromatography (HPLC). The calibration of SFN in rat plasma was done using a Jasco HPLC (Jasco PU 1580, UV-2075 Plus, Japan) system with a UV detector, and data was analysed using Jasco Borwin software. The separation was done using a reversed-phase C18 column (5 μm, 250×4.6 mm, MZ-Analytical, Germany) with an attached guard column (Shodex OHpak SB-G) at room temperature. The composition of the mobile phase was acetonitrile, methanol, and 1 % acetic acid (35:40:25 v/v) at a flow rate of 1 ml/min with an injection volume of 20 μl. The SFN eluent was monitored at 265 nm. The SFN analysis was adapted from a previously developed method with a slight modification [5]. Briefly, in 150 μl of fresh plasma, an aliquot of 150 μl of SFN solution was added. The 500 μl of extracting solvent methanol: acetonitrile (4: 1) was added to the above solution, vortexed (Spinex), and sonicated (Athena-ATS2-LCD) for 20 min. The precipitated plasma was separated by centrifuging at 15000 rpm at 4 °C for 20 min (Eppendorf-5424R). The supernatant was separated, filtered through a 0.2 μm syringe filter, and injected into HPLC [14].

### Animals

A total of 24 male Wistar rats (200-220 g) were procured from the National Institute of Biosciences, 1091/ABC/07/CPCSEA. The experimental protocol was approved by the institutional Animal ethics committee (IAEC) of Poona College of Pharmacy (IAEC no. 1703/PO/Re/S/01/CPCSEA), Bharti Vidyapeeth Deemed University. The protocol approval no. is CPCSEA/PCP/PCT22/2018-19, along with ARRIVE CPCSEA and ARRIVE guidelines (2.0 version). The care and use of experimental animals were as per CPCSEA guidelines. The sample size of *n* = 12 animals/group was selected. The randomization of animals in different groups was done by using the software graph prism tool. The observations were recorded by the observer, who was blind to the treatments. The animals were housed in 12 hour alternative light and dark cycles at 24 °C temperature and 55 % relative humidity. The animals were given access to standard food pellets and filtered water [16].

### *In vivo* pharmacokinetics study in rats

The pharmacokinetic study was conducted in 24 male Wistar rats where animals were divided into two groups (*n* = 12). Group I received pure SFN (20 mg/kg) aqueous suspension, and Group II received SFN-SSD (equivalent SFN 20 mg/kg) orally [16]. The 0.5 mL of blood was collected from a retro-orbital puncture under anaesthesia by thiopentone sodium (45 mg/kg) at 0.5, 1, 2, 4, 8, 12, 24, 6 and 48-hour time intervals into EDTA tubes. Plasma was separated by centrifuging at 10,000 rpm for 15 min at 4 °C and stored in a deep freezer (Thermoscientific-EXF24086V) at -20 °C till further analysis. The amount of SFN in plasma was analysed by the previously described HPLC method. The assessment of pharmacokinetic parameters such as area under the curve ((AUC (0 → 48 h)), elimination rate constant (*K*_el_)), maximum concentration (*C*_max_), time to peak drug concentration (*T*_max_), and half-life (*t*_1/2_) were calculated by noncompartmental analysis using PK Solver® software (version 2.1; Pharsight Co., Mountain View, CA, USA) [10,16].

### Statistical analysis

Student’s t-test was used for statistical analysis of a sample by one-way analysis of variance (ANOVA). The results were quoted as significant if *p* < 0.05. All data were given as mean ± standard deviation. (GraphPad Prism v8.0.2, USA).

## Results and discussion

### Saturation solubility study

**Figure 2.**
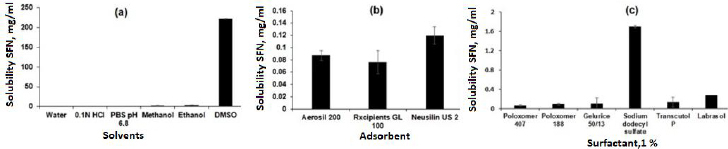
SFN solubility in different (a) solvents, (b) adsorbents and (c) surfactants

The saturation solubility was performed in different solvents to select the suitable solvent for SSD preparation. As shown in Figure 2a, the SFN showed very slight solubility in water (0.0288 ± 0.0017 mg/ml and the highest solubility was observed in DMSO (221.79 ± 45.26 mg/ml). [22,23]. The solubility order was DMSO > ethanol > methanol > 0.1 M HCl > acetate buffer pH 4.4 > phosphate buffer pH 6.8 > water, respectively. Further, the SFN showed insufficient solubility in common organic solvents such as methanol and ethanol. Considering the formulation aspect, DMSO and was selected for SSD preparation.

### Screening of adsorbents

The silica-based adsorbents such as Aerosil 200, Rxcipients GL 100, and NU2 were selected to screen the SFN solubility enhancements because of their advantages like high surface area, non-toxicity, and non-biodegradability [24]. As shown in Figure 2b, the NU2 showed the highest solubility of SFN (0.1198 ± 0.014 mg/ml) as compared to other adsorbents, which may be due to its surface features such as silanol functional groups responsible for the hydrogen bond with drug molecule and leads to improved solubility. Also, the NU2 is highly safe, and no adverse reactions were reported in the US Pharmacopoeia, National Formulary, and Japanese Pharmaceutical Codex [18,25]. Considering the formulation and solubility aspects, the NU2 was selected for SSD preparation.

### Screening of surfactant

Surfactants are chemical compounds that reduce surface tension or increase surface free energy. Besides that, surfactants have been widely used as wetting and solubilizing agents due to their ability to alter physical properties such as hydrophobicity and wetting properties. To select an appropriate surfactant, their ability to dissolve SFN was analysed in 1 % w/v solution of Poloxamer 407, Poloxamer 188, Gelucire 50/13, SDS, Transcutol P and Labrasol. As shown in Figure 2c, the SDS showed maximum solubility (1.7027 ± 0.1186 mg/ml) of SFN compared to others. The improved solubility is attributed to its higher hydrophilic lipophilic balance (HLB) value (40), micellar solubilization, wetting of the drug particles and decreasing the surface tension [26-29]. In this study, the SDS surfactant was selected to prepare SSD of SFN.

### Preparation and optimisation SFN-SSD

The SFN-SSD was prepared using a solvent evaporation technique and optimized for the concentration of adsorbent and surfactant by adopting 3^2^ factorial design using Design-Expert® software version 11, Stat-Ease, USA. A total of nine batches were prepared in accordance with the factorial design. The multiple regression analysis for the solubility of factorial batches revealed a fair fit (*R*^2^ = 0.460). The positive coefficient for both independent variables influencing the solubility of the SFN was given by equation (2):


(2)

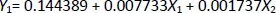



**Figure 3.**
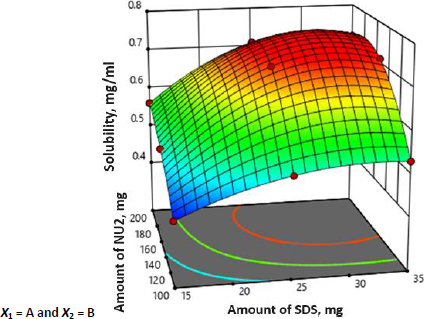
Response surface plot showing effect of factorial variables on SFN solubility

It was observed that the predicted *R*^2^ of 0.5846 is in reasonable agreement with the adjusted *R*^2^ of 0.7309. Further, the *P*-value and the model *F*-value less than 0.0500 and 11.86 implies the model is significant. Moreover, all batches were subjected to solubility analysis and results are shown in Table 2 and the surface response 3D plot is depicted in Figure 3.

The 3D surface response plot of the interaction between the NU2 and SDS was plotted with the response of SFN solubility. The surface plot showed that the adsorbent had a significant (*p* < 0.05) effect on the solubility until the concentration of 150 mg; after that, there were no significant increments in SFN solubility, which may be due to the adsorption SFN on NU2. Therefore, 150 mg of NU2 is taken as an optimised concentration. NU2 shows significant variation in effect with the surfactant concentration. The batches with higher concentration of the surfactant (25 mg), the adsorbent shows the highest effect compared to the batches with lower surfactant concentrations (15 mg). This may be because of the insufficient adsorption of the surfactant-drug molecule on NU2 at a lower concentration. Similarly, SDS with a larger amount (>25 mg) doesn’t show much difference; considering the cost of excipients and formulation efficiency, batch 5 was selected as an optimised batch and further evaluated as shown in Table 3.

### Yield and drug content

The dried weight of SFN-SSD was recorded to determine the practical yield. The samples were then dissolved in an appropriate amount of methanol using a cyclomixer and ultrasonicator. After making the necessary dilutions, the SFN content was measured at 265 nm with a spectrophotometer (UV-530; JASCO, Japan).

### Fourier transform infrared spectroscopy

**Figure 4.**
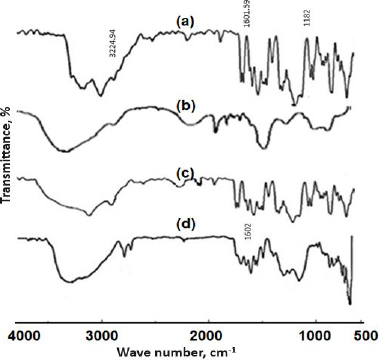
FTIR spectra of a - SFN, b - Neusilin US2, c - Physical mixture and d - SFN-SSD

The FTIR spectra of pure SFN, NU2, physical mixture and SFN-SSD are shown in Figure 4. The FT-IR spectrum of pure SFN showed unique bands at 3224.96 cm^-1^ (N-H stretching vibration of the NH group), 1601.59 cm^-1^ (C=O stretching vibration of the amide group) and 1182 cm^-1^ (C-F stretching) [30,31]. In the spectrum of the SFN-SSD, the SFN adsorbed at NU2 showed weaker C=O stretching of the amide at 1602 cm^-1^ due to interaction of Mg^+2^ and Al^+3^ with the carbonyl group of amides. Transfer of a lone pair of electrons to the metal ions leads to the conversion of -C=O- to -CO-, indicating a π-π* transition. The C-O bond is weaker compared to the C=O group, leading to the shifting of a peak. Further, the carboxylic group of SFN is bonded with the silanol group of NU2 through hydrogen bonding indicated in the form of a broad peak between 3000 and 2500 cm^-1^. Thus, H bonding could be responsible for the improvement in the solubility and dissolution rate of SFN [32,33].

### X-ray powder diffraction

**Figure 5.**
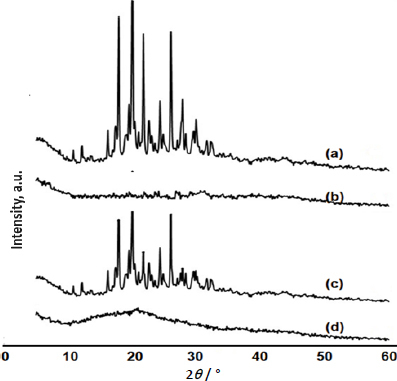
Powder X-ray diffraction patterns of a - SFN, b - Neusilin US2, c - physical mixture and d. SFN-SSD

The PXRD was performed to confirm the crystallinity of SFN in SSD. The X-ray diffraction patterns of the pure SFN, NU2, physical mixture and SFN-SSD are shown in Figure 5.

The pure SFN exhibited characteristic PXRD peaks at 2TTTTTTTTTT of 17.9, 20.03, 21.7 and 26.04°, indicating its crystalline nature as previously reported [34]. In the case of the PXRD patterns of SFN-SSD, the intensity of the characteristic peaks shown by the pure SFN disappeared, indicating the complete molecular dispersion of SFN in adsorbent matrices as an amorphous state [35]. The amorphous nature of SFN in SSD can be attributed to improvement in solubility.

### Scanning electronic microscopy

**Figure 6.**
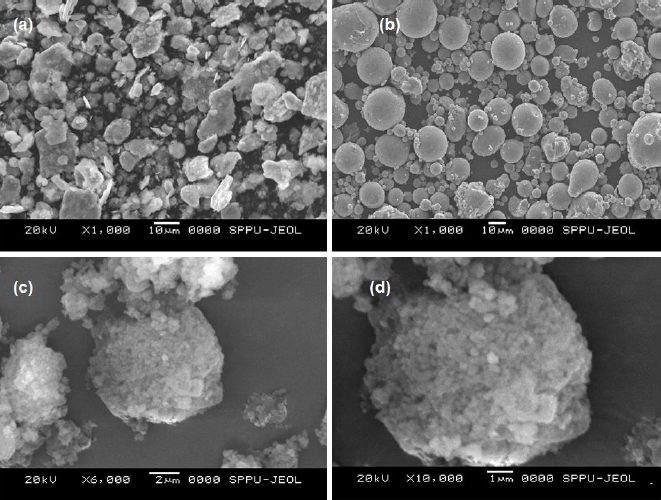
Scanning electron microscopy of a - SFN, b - Neusilin US2, c and d - SFN-SSD

The surface morphology of pure SFN, NU2 and SFN-SSD was studied using SEM, as shown in the Figure 6. The pure SFN showed irregular crystals with a plane surface, while NU2 showed smooth spherical, round-edged, non-uniform-sized amorphous particles. The SFN-SSD revealed the deposition of a fine microcrystal of SFN and surfactant on the surface of NU2 particles. The adsorption of an SFN on the NU2 surface leads to the formation of a rough surface of the adsorbent, indicating the formation SSD [36,37].

### Differential scanning calorimetry

**Figure 7.**
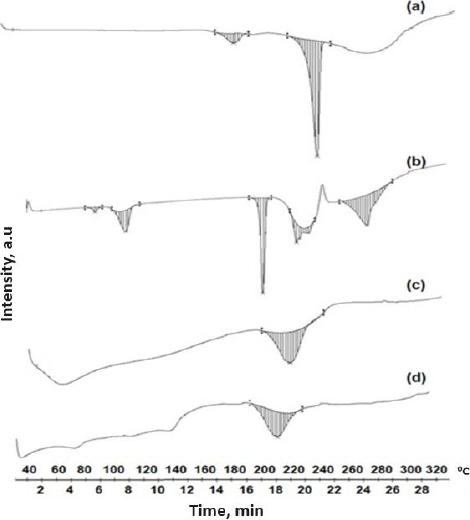
Differential scanning calorimetry of a - SFN, b - sodium dodecyl sulphate, c - Neusilin US2 and d - SFN-SSD

The thermal behaviour of SFN, NU2, and the SFN-SSD were studied using DSC. As shown in Figure 7, the SFN showed a sharp melting endotherm event at 236.39 °C with an enthalpy of fusion of -25.09 J/g, indicating crystalline nature. NU2 showed a broad melting peak at 212.84 °C (Δ*H* is -3.42 J/g), indicating its amorphous nature. The SFN-SSD showed a single broad peak at 210.16 °C with an enthalpy value of -2.72 J/g, indicating the NU2 peak with decreased melting point and enthalpy of fusion further indicates the incorporation of SFN into SSD. However, the SFN peak disappeared in SFN-SSD, indicating the conversion of SFN into the amorphous form [38]. The formation of amorphous SSD could result in an improvement in dissolution rate due to the solubilizing effect of a carrier, improved wetting, and a reduction in the crystallinity of the SFN [39,40].

### Evaluation of SFN-SSD and SFN immediate-release tablet

The SFN-SSD granules showed good flow properties such as bulk density of 0.393 g/ml, tapped density of 0.460 g/ml, Carr’s compressibility index of 14.5 %, Hausner’s Ratio of 1.17 and angle of repose of 27.52°. As shown in Table 4, both tablets are within the pharmacopeial limit of hardness, thickness, diameter, weight variation and friability. The disintegration time was in the pharmacopeial immediate release tablet limit, which may be due to the capillary action that facilitates the absorption of water, leading to higher water solubility. Moreover, the use of MCC induces faster disintegration.

### *In vitro* dissolution studies

**Figure 8.**
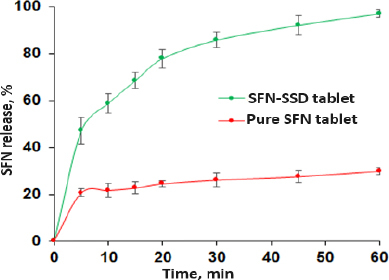
*In vitro* dissolution profiles of SFN-SSD and pure SFN tablets

The *in vitro* release profiles of SFN-SSD and pure SFN tablets were shown in Figure 8. The SFN-SSD tablet showed an initial burst release due to micellar solubility of SDS with a cumulative release of 97.13 % in 60 min. This might be the reason for the rapid diffusion of SFN, which was absorbed on the surface of micelles, or due to the disruption of the micellar structure due to pH or the presence of ionic molecules in dissolution media. Pure SFN tablets showed 29.93 % release at the same time, indicating poor aqueous solubility of SFN. Further, the rapid release of SFN from the SFN-SSD tablet was due to the improved aqueous solubility of SFN by converting crystalline to amorphous form with a high energy state in SSD. As reported in the literature, the basic mechanism of drug dissolution from silica-based adsorbents is stronger interactions between the silica and water than between the silica and drug due to hydrogen bonding. In SSD, SFN formed hydrogen bonds with NU2. In a dissolution medium, the water molecules remove the drug from the adsorbent, and the drug gets solubilized in the sheath layer of the adsorbent [17,18]. The stronger hydrogen bond formation between NU2 and water molecules causes the cleavage of the weaker hydrogen bond between NU2-SFN. The SFN release from SSD follows first-order kinetics (*R*^2^ = 0.9839), indicating the drug release depends on the drug concentration. This may be because the drug present on the surface of the adsorbent getting dissolved leads to a decrease in concentration. However, pure SFN tablets showed < 30 % release in 1 hour. According to the US FDA, the dissolution of SFN formulations is evaluated with 0.1 M HCl containing 1 % sodium lauryl sulphate (SLS) [40]. In this study, 1 % SLS was not used in the dissolution medium because our major effort was to increase the aqueous solubility and dissolution properties of SFN using surface solid dispersion [15,41].

### In vivo pharmacokinetic studies in rats

**Figure 9.**
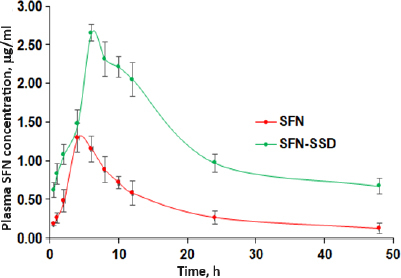
Plasma concentration-time profiles of SFN from SFN-SSD and pure SNF in rats after oral administration. Data are presented as the mean ± standard error (*n*= 3)

The SFN-SSD was prepared to improve the solubility and bioavailability of SFN. The plasma levels after oral administration of SFN-SSD were compared with pure SFN. The mean plasma SFN concentration (cg/ml) after oral administration of a single dose of the SFN-SSD and pure SFN with time (h) are shown in Figure 9.

The relevant pharmacokinetics parameters derived by noncompartmental analysis are given in Table 5. The SFN-SSD showed 2- and 6.5-folds improvement in *C*_max_ (*p* < 0.01) and AUC_0-t_ as compared to pure SFN due to faster drug release at the site of absorption from the SFN-SSD. The improved SFN concentration in the blood may be because of an enhancement in the aqueous solubility of SFN and subsequent absorption [38,41].

## Conclusion

This study demonstrates the utility of SSD in improving the biopharmaceutical properties of SFN. The SFN-SSD was successfully prepared using a solvent evaporation method with NU2 as the adsorbent and SDS as the surfactant and optimized using Design Expert 11. The SFN-SSD reflected improved oral bioavailability compared to the pure form of SFN due to faster drug release at the absorption site. The study concluded that SSD could be a scalable formulation approach and a more industry-friendly technology to improve the biopharmaceutical properties of SFN.

## Supplementary material

Data for DOE formulation optimization are available electronically on the journal's website page: https://pub.iapchem.org/ojs/index.php/admet/article/view/2338, or from the corresponding author upon request.



## Figures and Tables

**Table 1.** Independent variables and levels for the optimization by 3^2^ full factorial design

Independent variables	Levels			Dependent variable
	-1	0	+1	Solubility of SFN
	Amount, mg			
Concentration of SDS	15	25	35	
Concentration of NU2	100	150	200	

**Table 2.** Composition of SFN-SSD and SFN immediate release tablet

Batches	Amount, mg		Solubility*, mg/ml
	SDS	NU2	
1	15	100	0.424 ± 0.019
2	25	100	0.513 ± 0.022
3	35	100	0.531 ± 0.032
4	15	150	0.513 ± 0.029
5	25	150	0.703 ± 0.024
6	35	150	0.711 ± 0.024
7	15	200	0.561 ± 0.032
8	25	200	0.713 ± 0.054
9	35	200	0.719 ± 0.038

*All the determinations were performed in triplicate and values are expressed as mean ± SD (n = 3)

**Table 3.** Composition of the SFN-SSD and pure SFN tablet

Ingredients	Amount, mg	
	SFN-SSD tablet	Pure SFN tablet
SFN-SSD	456.5 (equivalent to 100 mg SFN)	100.0 (SFN)
Croscarmellose Sodium	20.0	20.0
MCC	0.0	356.5
PVP K30	10.0	10.0
Magnesium stearate	5.0	5.0
Talc	5.0	5.0
Total weight	506.5	506.5

All the determinations were performed in triplicate

**Table 4.** Evaluation of SFN-SSD and SFN immediate release tablet

Parameters	SFN-SSD tablet (B1)	Pure SFN tablet (B2)
Hardness, kg/cm^2^	4.21 ± 0.36	4.73 ± 0.15
Thickness, mm	5.53 ± 0.15**	5.83 ± 0.08
Diameter, mm	12.03 ± 0.06	12.00 ± 0.00
Weight variation	Passes (average weight 507.5 mg)**	Passes (average weight 507.1 mg)
Friability, %	0.39 ± 0.06	0.28 ± 0.36
Disintegration, min	2.87 ± 0.35*	2.27 ± 0.42

All the determinations are performed in triplicate, and values are expressed as the mean data are mean ± SD (*n* = 3)

*p ≤ 0.1 (not statistically significant)

**p ≤ 0.01 (statistically significant)

**Table 5:** Oral pharmacokinetic parameters of SFN-SDD and pure SFN in rats

Parameters	SFN	SFN-SSD
*C*_max_ / μg mL^-1^	1.318 ± 0.186	2.655 ± 0.406^*^
*t*_max_ / h	4.661 ± 1.155	3.0 ± 0.0
AUC, μg mL^-1^	9.381 ± 4.966	59.686 ± 7.858*
*K*_el_ / h^-1^	0.0488 ± 0.0120	0.0336 ± 0.0075
*t*_1/2_ / h	14.83 ± 3.84	21.40 ± 5.19
*Cl F*^-1^ / (mg/h)/g	0.190 ± 0.056	0.049 ± 0.005
*V F*^-1^ / mg (μg ml^-1^)^-1^	3.889 ± 0.439	1.515 ± 0.209

Each value represents the mean ± standard deviation (*n* = 3)

^*^p < 0.05; *Cl* - clearance rate; *V* - volume of distribution

## List of abbreviations

DMSO: dimethyl sulfoxide
DDW: double distilled water
DSC: differential scanning calorimetry
DOE: design of experiment
FTIR: Fourier transform-infrared spectroscopy,
IPA: isopropyl alcohol
IP: Indian pharmacopoeia
MAS: magnesium aluminometasilicate
PDGFR: platelet-derived growth factor receptor
PXRD: powder X-ray powder diffraction
SEM: scanning electronic microscopy
SFN: sorafenib tosylate
SSD: surface solid dispersion
SDS: sodium dodecyl sulphate
