# Supplementary material for: Investigation of magnesium aluminometasilicate (Neusilin US2) based surface solid dispersion of sorafenib tosylate using QbD approach: In vitro and in vivo pharmacokinetic study
Source: ADMET DMPK. 2024 Aug 9;12(4):687–702. doi: 10.5599/admet.2338 (PMC11517514; doi:10.5599/admet.2338)
Supplement: Supplementary file 2 [file ADMET-12-2338-S1.docx]

*ADMET & DMPK 12(4) (2020) S5-S6*

*
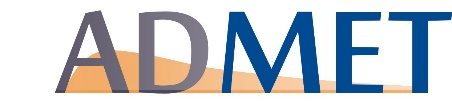
***Open Access : ISSN : 1848-7718**[***http://www.pub.iapchem.org/ojs/index.php/admet/index***](http://www.pub.iapchem.org/ojs/index.php/admet/index)

Supplementary material to

Investigation of magnesium aluminometasilicate (Neusilin US2) based surface solid dispersion of sorafenib tosylate using QbD approach: *In vitro* and *in vivo* pharmacokinetic study

Bijoy Kumar Panda^1^, Bothiraja Chellampillai^2^, Sharad Ghodake^3^, Ashwin J. Mali^3^ and Ravindra Kamble^3^

^1^Department of Pharmacy Practice, Krishna Institute of Pharmacy, Krishna Vishwa Vidyapeeth (Deemed to be University), Karad, Maharashtra, India

^2^Department of Pharmaceutics, Goa College of Pharmacy, Goa University, Panaji, Goa, India

^3^Department of Pharmaceutics, Poona College of Pharmacy, Bharati Vidyapeeth (Deemed to be University), Pune, Maharashtra, India

ADMET & DMPK**12(4)** (2024) 687-602; <https://doi.org/10.5599/admet.2338>

Data for optimization of formulation by DOE

ANOVA for Linear model

Response 1: Solubility

| Source | Sum of squares | df | Mean Square | *F*-value | *p*-value |  |
| --- | --- | --- | --- | --- | --- | --- |
| Model | 0.0811 | 2 | 0.0406 | 11.86 | 0.0082 | significant |
| A-SDS | 0.0359 | 1 | 0.0359 | 10.49 | 0.0177 |  |
| B-NU2 | 0.0452 | 1 | 0.0452 | 13.23 | 0.0109 |  |
| Residual | 0.0205 | 6 | 0.0034 |  |  |  |
| Cor Total | 0.1016 | 8 |  |  |  |  |

Factor coding is **Coded**

Sum of squares is **Type III - Partial**

The Model F-value of 11.86 implies the model is significant. There is only a 0.82 % chance that an *F*-value this large could occur due to noise.

*P*-values less than 0.0500 indicate model terms are significant. In this case A, B are significant model terms. Values greater than 0.1000 indicate the model terms are not significant. If there are many insignificant model terms (not counting those required to support hierarchy), model reduction may improve your model.

Fit statistics

| Std. Dev. | 0.0585 | *R*² | 0.7982 |
| --- | --- | --- | --- |
| Mean | 0.5982 | Adjusted *R*² | 0.7309 |
| C.V., % | 9.77 | Predicted *R*² | 0.5846 |
|  |  | Adeq Precision | 9.7256 |

The predicted *R*² of 0.5846 is in reasonable agreement with the adjusted *R*² of 0.7309; *i.e.* the difference is less than 0.2.

Adeq Precision measures the signal to noise ratio. A ratio greater than 4 is desirable. Your ratio of 9.726 indicates an adequate signal. This model can be used to navigate the design space.

Model comparison statistics

| PRESS | 0.0422 |
| --- | --- |
| -2 log likelihood | -29.21 |
| BIC | -22.62 |
| AICc | -18.41 |

Coefficients in terms of coded factors

| Factor | Coefficient estimate | df | Standard error | 95 % CI Low | 95 % CI High | VIF |
| --- | --- | --- | --- | --- | --- | --- |
| Intercept | 0.5982 | 1 | 0.0195 | 0.5505 | 0.6459 |  |
| A-SDS | 0.0773 | 1 | 0.0239 | 0.0189 | 0.1357 | 1.0000 |
| B-NU2 | 0.0868 | 1 | 0.0239 | 0.0284 | 0.1452 | 1.0000 |

The coefficient estimate represents the expected change in response per unit change in factor value when all remaining factors are held constant. The intercept in an orthogonal design is the overall average response of all the runs. The coefficients are adjustments around that average based on the factor settings. When the factors are orthogonal the VIFs are 1; VIFs greater than 1 indicate multi-colinearity, the higher the VIF the more severe the correlation of factors. As a rough rule, VIFs less than 10 are tolerable.

Final equation in terms of coded factors

| Solubility | = |
| --- | --- |
| +0.5982 |  |
| +0.0773 | A |
| +0.0868 | B |

The equation in terms of coded factors can be used to make predictions about the response for given levels of each factor. By default, the high levels of the factors are coded as +1 and the low levels are coded as -1. The coded equation is useful for identifying the relative impact of the factors by comparing the factor coefficients.

Final equation in terms of actual factors

| Solubility | = |
| --- | --- |
| +0.144389 |  |
| +0.007733 | SDS |
| +0.001737 | NU2 |

The equation in terms of actual factors can be used to make predictions about the response for given levels of each factor. Here, the levels should be specified in the original units for each factor. This equation should not be used to determine the relative impact of each factor because the coefficients are scaled to accommodate the units of each factor and the intercept is not at the center of the design space.
